# Making encounters count: Healthcare professionals’ experiences of providing care in Norwegian sexual assault centres

**DOI:** 10.1177/17455057261463284

**Published:** 2026-08-18

**Authors:** Siri Haugan, Cecilie Therese Hagemann, Kjersti Alsaker, Marianne Kjelsvik

**Affiliations:** 1 Department of Health Sciences in Aalesund, Faculty of Medicine and Health Sciences, 620066 Norwegian University of Science and Technology (NTNU), Aalesund, Norway; 2 Department of Public Health and Nursing, Faculty of Medicine and Health Sciences, 8018 Norwegian University of Science and Technology (NTNU), Trondheim, Norway; 3 Department of Clinical and Molecular Medicine, Faculty of Medicine and Health Sciences, 8018 Norwegian University of Science and Technology (NTNU), Trondheim, Norway; 4 Department of Obstetrics and Gynecology, St. Olavs Hospital, Trondheim University hospital, Trondheim, Norway; 5 Department of Health and Caring Sciences, Western Norway University of Applied Sciences (HVL), Bergen, Norway; 6 322041 Division of Health and Social Sciences, NORCE Norwegian Research Centre AS, Bergen, Norway

**Keywords:** care after sexual assault, forensic examination, medico-legal practice, trauma-informed care, emotional labor

## Abstract

**Background:**

Although the negative health consequences of sexual assault are well documented, less is known about how healthcare experience and navigate their clinical and medico-legal roles when providing care in the immediate aftermath of an assault. Understanding these processes is important for strengthening care practices within sexual assault centres (SACs).

**Objectives:**

To explore how healthcare personnel (HCP) at Norwegian SACs understand, reflect on, and navigate their work during acute sexual assault consultations, to better understand the clinical, relational, and medico-legal dimensions of this care.

**Design:**

Qualitative study based on focus groups and reflexive thematic analysis.

**Methods:**

Four focus group interviews were conducted with physicians and nurses from four SACs across Norway. Interviews lasted for 75–100 minutes and explored clinical decision-making, emotional experiences, and perceptions of the SAC role. Data was analysed using Braun and Clarke’s reflexive thematic analysis, which emphasizes the active, interpretive role of researchers and is well suited to examining how professionals construct meaning in complex care contexts.

**Results:**

Three overarching themes were identified. (1) *A practice to believe in – professional purpose despite limited training and lack of institutional recognition*. Healthcare personnel described their work as meaningful and socially important, despite limited training and a lack of institutional recognition. (2) *Navigating medico-legal demands while providing care in vulnerable encounters*. Healthcare personnel reflected on balancing forensic responsibilities with patients’ needs for safety, autonomy, and dignity. (3) *The need to reset – carrying the unsolved*. HCP described the emotional residue of SAC work, the lingering impact of unresolved cases, and the role of collegial support and personal strategies in sustaining professional endurance.

**Conclusion:**

Healthcare professionals in Norwegian SACs navigate complex clinical, medico-legal, and emotional demands when providing care after sexual assault. The findings highlight tensions between care and forensic responsibilities and point to the need for strengthened training and organizational support. Greater attention to these dimensions may improve how care is delivered in SAC settings.

## Plain language summary

Although the negative health consequences of sexual assault are well documented, less is known about how healthcare experience and navigate their clinical and medico-legal roles when providing care in the immediate aftermath of an assault. Understanding these processes is important for strengthening care practices within sexual assault centres (SACs). To explore how healthcare personnel (HCP) at Norwegian SACs understand, reflect on, and navigate their work during acute sexual assault consultations, to better understand the clinical, relational, and medico-legal dimensions of this care. Qualitative study based on focus groups and reflexive thematic analysis. Four focus group interviews were conducted with physicians and nurses from four SACs across Norway. Interviews lasted for 75-100 minutes and explored clinical decision-making, emotional experiences, and perceptions of the SAC role. Data was analysed using Braun and Clarke’s reflexive thematic analysis, which emphasizes the active, interpretive role of researchers and is well suited to examining how professionals construct meaning in complex care contexts. Three overarching themes were identified. (1) *A practice to believe in- professional purpose despite limited training and lack of institutional recognition.* Healthcare personnel described their work as meaningful and socially important, despite limited training and a lack of institutional recognition. (2) *Navigating medico-legal demands while providing care in vulnerable encounters.* Healthcare personnel reflected on balancing forensic responsibilities with patients needs for safety, autonomy, and dignity. (3) *The need to reset-carrying the unsolved*. HCP described the emotional residue of SAC work, the lingering impact of unresolved cases, and the role of collegial support and personal strategies in sustaining professional endurance. Healthcare professionals in Norwegian SACs navigate complex clinical, medico-legal, and emotional demands.

## Introduction

Healthcare personnel (HCP) at Norway’s 23 sexual assault centres (SACs) play a vital role in the immediate and follow-up care of sexual assault survivors.^1–3^ Each year, around 2,450 individuals, 95% of them women, seek support at these centres.^4^ For many sexual assault survivors, HCP are the first point of contact and disclosure.^2^ SACs in the Nordic countries are situated within healthcare institutions, providing 24/7 care to survivors of sexual assault, including forensic medical examination, psychosocial support, and follow-up services, regardless of whether the assault has been reported to the police.^5^ Suspected assailants in police-reported cases are usually examined outside Norwegian SACs, usually by the police itself or by a physician elsewhere.

This study was conducted in Norway, a country often highlighted for its strong welfare system and commitment to social and gender equality.^6^ Nevertheless, sexual violence remains a significant public health concern.^7^ A recent population-based study found that 22% of women and 3% of men have experienced rape in their lifetime. Yet, only one in five cases are reported to the police, often due to fears of not being believed or concerns about social consequences, and only 10% of survivors seek acute help from healthcare services.^5,7^ These findings suggest that sexual violence remains highly stigmatized, even in a society characterized by progressive gender norms.^4^ Within this context, SACs play a critical role as one of the few accessible points of support and care for survivors in the immediate aftermath of an assault. Recent national recommendations have proposed strengthening competence requirements, training, and working conditions for staff in these services, highlighting that high-quality care depends on well-supported and skilled professionals.^4^

These services offer multidimensional consultations that are tailored to the characteristics of the assault and the patient’s individual needs and preferences.^8,9^ Consultations at SACs include assessing the detailed history of the recent assault, the patient’s medical history, and the personal context including relevant sociodemographic information (e.g., age, living situation, employment), all of which are to be documented in the medical record. Caswell et al.^10^ highlighted the importance of fostering patient empowerment, restoring a sense of control, and ensuring HCP communicate with clarity and empathy, before and during examination procedures.^8,11^

Internationally, the first SACs or one-stop centres were established in the United States and Australia in the 1970s,^12,13^ and similar centres have since been implemented worldwide to provide integrated medical, psychological, and legal support.^8,13–15^ In Europe, the first sexual assault centre was established in Ireland, where the Sexual Assault Treatment Unit (SATU) at the Rotunda Hospital in Dublin opened in 1985,^13^ followed by the first Norwegian centre in Oslo in 1986.^4^ International research, including large-scale evaluations such as the MeSARCh^16^ project in England, has shown that sexual assault referral centres are often organised as multidisciplinary, inter-agency services involving healthcare, advocacy, and criminal justice sectors. In Norway, SACs are similarly embedded within the healthcare system; however, the organization of services is shaped by the country’s geography, with many small and locally based centers. This reflects a broader tradition of decentralized healthcare and strong primary care involvement. Such structural features may influence professional roles, resource availability, and care practices, highlighting the importance of context-specific research.^1^

In this study, we draw on the concept of person-centered care^17,18^ to describe a relational approach that recognizes the individual as a whole person rather than primarily as a patient, emphasizing personhood, dignity, autonomy, lived experience, and the co-creation of care through meaningful relationships.^17^ Within sexual and reproductive health, person-centred care has been highlighted as central to high-quality services, foregrounding respect, communication, and emotional support.^18^ This aligns with trauma-informed care frameworks,^19^ which emphasize recognizing the impact of trauma and providing care that supports safety, trust, and patient empowerment. Together, these frameworks provide important guiding principles for sexual assault care. Building on these trauma-informed principles, care is often described as being provided through national services with clear interagency guidelines, ensuring accessible and trauma-informed support for all individuals, whether or not they choose to report the assault to the police, while also emphasizing prevention, education, and ongoing staff support.^11,13^

Consultations at SACs are characterized by multiple and often conflicting demands, as HCP must provide emotional care and safety for patients while simultaneously conducting detailed medical and gynaecological examinations and collecting thorough forensic specimens.^1,8,20,21^ These encounters take place under pressure and in high-stakes environments, often involving patients and their next of kin in states of shock, distress, or confusion.^1,21^ In this context, clinicians are required to balance their legal and procedural responsibilities with the therapeutic needs of individuals who are often highly vulnerable.^21–24^ Sexual assault can have profound and long-lasting consequences for survivors’ physical, psychological, and relational health.^25^ When disclosures are dismissed or remain unheard, the impacts are devastating, reinforcing silence and exacerbating trauma.^26,27^

This tension between care and forensic responsibility has been critically examined by Mulla,^28^ who conceptualizes it as “the violence of care,” where institutional and procedural demands may inadvertently reproduce harm to patients. From this perspective, HCP operate within a system where therapeutic intentions coexist with practices that may be experienced as invasive or disempowering. Responses to sexual assault must therefore acknowledge the traumatizing and sexualized nature of such violence, challenge cultural stereotypes that minimize its seriousness, and recognize that psychological violence often co-occurs, compounding shame and hindering survivors’ ability to articulate their distress.^29,30^ Research further highlights that sexual trauma exerts a unique and enduring impact on symptom severity compared to nonsexual trauma.^31^ Within healthcare, even clinical encounters can lead to secondary victimization if examinations are not guided by clear and compassionate communication, informed consent, and sensitivity to patients’ vulnerabilities.^8,32^ Patients’ willingness to undergo procedures such as pelvic examinations depends on a transparent understanding of their purpose and conditions, which is essential to promote trust and safety in care.^33^

Norwegian SACs are publicly funded, integrated into the public healthcare system and are staffed by multidisciplinary teams, although the organization and availability of resources vary across centers due to differences in staffing and access to forensic expertise.^1^ In Norwegian SACs, physicians hold primary medico-legal responsibility, while nurses play a central role in patient reception, psychosocial support, evidence handling, and injury documentation. Although roles overlap, responsibilities differ between the SACs, particularly in medico-legal examinations and forensic documentation. Nurses contribute with key procedural expertise and continuity of care. Although national training programs aim to promote standardized competence, there are no formal national competence requirements for SAC staff, which results in considerable variation in training, resources, and structure of the psychosocial follow-up for the patients.^1^ These differences in professional roles and organizational conditions may shape how healthcare professionals understand and navigate their responsibilities in acute consultations.^1^ Several contextual challenges have been faced by HCP at the sexual assault centre in the Norwegian context. SAC duties are often performed alongside other clinical roles, which results in limited routine experience, inconsistent supervision, and insufficient training. Combined with high forensic demands and minimal preparation in evidence collection and documentation, these structural constraints may increase the risk of stress, uncertainty, burnout, and professional vulnerability.^1^

Several qualitative studies have examined the experiences of HCP working in SACs, particularly in their encounters with adult patients. However, much of this research originates from the United States and the United Kingdom, where Sexual Assault Nurse Examiners (SANEs) have provided valuable insights into both the clinical and emotional dimensions of their work.^2,21,34–37^ A consistent theme across these studies is the relational challenge of building trust with patients while balancing compassionate care with legal and forensic responsibilities. SANEs have emphasized that their primary responsibility is patient care, initiating healing, providing emotional support, and ensuring patients feel neither judged nor blamed, whilst medico-legal procedures remain secondary.^12,21,35–37^ In contrast, Mulla^28^ draws attention to the structural tensions embedded in forensic care, where medico-legal demands may shape and at times limit patient-centred approaches.

Despite this body of international research, little is known about how HCP in Nordic SACs experience and make sense of their clinical and emotional work during acute consultations, involving only patients who have experienced sexual assault. The aim of this study was therefore to examine how HCP in Norwegian SACs reflect on and navigate acute consultations with sexual assault survivors, with particular attention to the interplay between the forensic, medical, and emotional dimensions of care.

## Methods

### Design

This study employed an exploratory qualitative design using focus group interviews and reflexive thematic analysis.^38^ Focus groups were chosen to explicitly utilize interaction between participants as a source of data, enabling exploration not only of what participants think, but how and why these understandings are constructed. Attention was given to encouraging dialogue among participants, allowing professional meanings to be explored and shaped through collective discussion.^39^ The study is reported in accordance with the Consolidated Criteria for Reporting Qualitative Research (COREQ) 32-item checklist to enhance transparency in study design, data collection and researcher reflexivity.^40^ Although reporting was informed by COREQ, the analytic approach was guided by the principles of reflexive thematic analysis, where concepts such as data saturation and coding reliability are not treated as indicators of rigour.^38,41^

### Recruitment

Participants were recruited from four SACs in various geographic regions in Norway. Recruitment was facilitated by the local SAC leaders, who informed HCP with clinical experience from SAC consultations about the study. Information about the project was disseminated through multiple channels, including written information sheets distributed by email and oral presentations at staff meetings and professional gatherings. Participation was voluntary, and no incentives were offered. HCP who expressed interest received written and oral information about the study and provided written informed consent prior to participation.

### Participants

Participants were eligible for inclusion if they had experience conducting acute SAC consultations, including medical, forensic, and psychosocial aspects of care; some also had experience with psychosocial follow-up consultations. No specific exclusion criteria were applied beyond lack of relevant clinical experience. Participants were distributed across four interprofessional focus groups with varying size and composition, reflecting differences in staffing across centers (Table 1).The sample included nurses (RN) and physicians (PH) with varying levels of clinical experience and representing both urban and rural SAC settings. The final sample (no drop out) consisted of physicians (n = 12), nurses (n = 15), and one medical student (MS) (n = 1). Participants were predominantly women (n = 26), with two men (n = 2), and ranged in age from 26 to 61 years. A detailed overview of the composition of each focus group, including number of participants, gender distribution, age, years of clinical experience, and professional background, is presented in Table 1.

**Table 1.** Overview of the composition of each focus group.

Group no.	No. of participants	Female/Male	Age mean (range)	Years of experience mean (range)	Professional composition: Physicians (PH), nurses (RN), Medical student (MS)
1	12	11/1	35.4 (29-48)	4.3 (1-17)	PH (9), RN (3)
2	5	5/0	35.6 (28-44)	4.8 (2- 8)	PH (1), RN (4)
3	7	7/0	43.1 (28-61)	5.9 (1-14)	PH (1), RN (5), MS (1)
4	4	3/1	39.5 (26-52)	7.0 (3-11)	PH (1), RN (3)
Total	28	26/2	26-61	1-17	PH (12), RN (15), MS (1)

### Data collection

Data was collected through four focus group interviews conducted between June 2022 and November 2023, allowing participants to reflect on and discuss shared professional experiences. The interviews were arranged by the first author (SH) and took place at locations convenient for the participants, at the clinic. Each focus group lasted for between 75 and 100 minutes. The interviews were moderated by the first author (SH), three were co-moderated by the fourth author (MK), and one by the third author (KA). No non-participants were present during the interviews. A semi-structured interview guide was developed to support an exploratory approach, informed by relevant literature and discussions within the research team. Open-ended questions included, for example: “Can you describe what the consultations at the SACs are like?” and “What motivates you to work at the SAC?” The questions were not designed to evaluate predefined frameworks, but to explore participants’ experiences and reflections. The interviews were audio-recorded and transcribed verbatim by SH and MK.

### Data analysis

Data analysis began concurrently with data collection and followed Braun and Clarke’s six-phase reflexive thematic analysis (TA) process: familiarization with the data, generating initial codes, searching for themes, reviewing themes, defining and naming themes, and producing the report.^38^ TA was chosen because it provides a systematic yet flexible approach to identifying patterns of meaning across qualitative material while allowing for interpretation of the complex interplay between individual experiences, professional practices, and contextual factors.^38,41,42^ The analysis was reflexive and iterative, involving ongoing discussions and movement back and forth between data and interpretation to refine and confirm themes. All authors were involved throughout the analytic process, from early analytic discussions to later theme development. SH, KA, and MK read transcripts, while CTH participated in the analytic process. The aim was not to establish a single truth but to provide a rich, grounded interpretation of participants’ experiences of working with patients at SACs.

### Ethical considerations

This study was designed in accordance with the Helsinki Declaration^43^ and received approval from the Norwegian Centre for Research Data (SIKT 97788). Furthermore, permission to conduct the study was obtained from the head clinical manager of each SAC. All participants provided written informed consent. In addition, all participating HCP were reminded of their duty of confidentiality and instructed not to disclose any identifiable patient information during the interviews. As participants were healthcare professionals with experience in this field, no formal follow-up support was arranged. Participants were considered able to access support through their usual professional or workplace resources if needed.

## Results

The analyses identified three overarching themes describing how HCP found professional meaning despite limited training and support, balanced medico-legal and relational demands in vulnerable encounters and managed the emotional impact of sexual assault care over time (Table 2).

**Table 2.** Overview of themes, subthemes, and central ideas of each theme.

Themes	Subthemes	The central ideas of the theme
A practice to believe in – professional purpose despite limited training and a lack of institutional recognition	Contributing to something greater Restoring patients’ dignity Individual stories – an important source of motivation Organizational conditions further contributed to a sense of vulnerability	HPC describe their work as deeply meaningful and motivated by the difference they can make for patients, yet they operate with limited training, unclear structures, and low institutional recognition.
Navigating medico-legal demands while providing care in vulnerable encounters	Double responsibility Balancing power and responsibility Provide care on the patient’s terms	HCP constantly navigated the tension between forensic accuracy and care. Whilst they were required to collect evidence and ask intrusive questions, they simultaneously sought to protect patients’ dignity and autonomy but found emotional safety to be demanding.
The need to reset – carrying the unsolved	Resetting themselves The emotional transition- rapidly shift from one demanding situation to another Importance of support and recognition Juggling different professional roles	HCP described the emotional weight of SAC care and the need to continuously “reset” themselves when moving between demanding clinical contexts, with unresolved cases and lingering impressions often following them beyond shifts. Collegial support, shared reflection, and individual strategies were essential to sustain emotional endurance and maintain compassionate care over time.

### A practice to believe in – Professional purpose despite limited training and a lack of institutional recognition

This theme captures how HCP found meaning and motivation by framing their work as part of a broader societal mission. Contributing both to patients’ recovery and to the legal process gave their work a sense of purpose that often outweighed the challenges they faced. Many described providing care after sexual assault as work that they believed in, experienced as meaningful both personally and professionally, and understood as a central societal responsibility that allowed them to **contribute to something larger than themselves**. This sense of purpose fostered a sense of pride and belonging, as reflected by one nurse: “It is important to me that we have a well-functioning SAC. I think the legal part is very important – it is important work, important work for the society.”

Forensic sampling and documentation were described as carrying both gravity and uncertainty yet were viewed as core responsibilities towards the patients and society. HCP expressed a strong concern about making mistakes, as the consequences were perceived as significant, not only for patients seeking justice but also to avoid the risk of wrongful accusation. This concern reflected an awareness of responsibility not only towards patients but also towards individuals who might later be identified as alleged perpetrators, underscoring the moral weight attached to forensic accuracy. Being part of a process that could lead to accountability and legal outcomes reinforced their sense of purpose, as one nurse described: “It is good to read in the newspaper … that someone went to court, and then there was a verdict. To be part of that; we did it, what we did mattered.” Seeing patients strengthened through the encounter and knowing that their efforts could make a difference were central sources of motivation. At the same time, HCP described how it could feel frustrating when patients declined forensic sampling or further examinations, highlighting a tension between respecting patients’ choices and their own sense of responsibility towards the legal process, as one of the nurses expressed: “When you have arranged everything, but then the patient just wants to go home.”

**Helping patients regain a sense of dignity** emerged as a particularly important source of motivation. HCP described how witnessing patients arrive distressed and leave with greater strength and self-worth affirmed their sense of professional purpose. As one nurse explained:

It is when you see that they enter completely collapsed, just crying and crying and crying. And then they can walk out the door with their head held high and their back straight. Then I feel that I have done a good job. You can see it in them. But of course**,** it’s not always something you can do. But that is the motivation in it.

Small but attentive relational acts, such as creating a calm environment, communicating availability, and adapting to each patient’s pace, were described as central to this work. These practices were experienced as the core of a professional approach that they believed in, and as a source of pride and meaning. Several HCP noted that the most serious cases often reinforced their motivation and shaped how meaningful they experienced their work, with clearer cases described as easier to engage with, and how these **individual stories was a source for motivation,** as expressed by one physician: “I feel that the job is most meaningful when it is a really serious case – when it is clear that someone needs help.” This did not imply less care for others; rather, the sense that someone was in urgent need strengthened their feeling of making an important contribution. At the same time, providing care was experienced as more challenging when patients had fragmented or absent memories of the assault. HCP described uncertainty about how to ask questions without increasing patients’ feelings of inadequacy or doubt. As one physician explained: “We cannot check whether someone has been assaulted. We don’t have that kind of possibility.” This uncertainty could make emotional engagement more difficult and challenge how HCP navigated the encounter. Nevertheless, HCP emphasized that care should never depend on whether the patient had a clear history or not, as expressed by one of the nurses:

It is not up to us to decide whether it was an assault or not, that is not what matters in that moment. They come to us regardless – and we are there to receive them, and they must be cared for no matter what. We must never forget that.

Despite the complexity of these encounters, HCP described that their ability to manage them had developed largely through experience rather than formal preparation. Structured training, supervision, and follow-up were described as limited, which meant that much competence was acquired informally through informal collegial support and repeated exposure. One nurse described the inadequacy of formal training:

So, when you get a message that there is an SA [sexual assault] patient coming in … and you have had only a two-hour course, that is far too little, considering everything you are supposed to know. You are thrown into something that is serious, when you stand there, basically just as a – yes, you get a bit shaky.

**Organizational conditions further contributed to a sense of vulnerability**. This might involve unpredictable shift schedules, unclear responsibilities, and the fact that SAC work was often an additional duty alongside other clinical roles, all of which reinforced uncertainty. Several HCP described care at SACs as undervalued within the healthcare system and perceived as being “at the bottom of the hierarchy”. As one nurse stated: “There’s a big difference between being a heart surgery patient and being a rape victim.” The practical realities of SAC work further reinforced this sense of fragility. In this context, fragility referred to a practice characterized by limited continuity, unstable routines, and insufficient structural support for developing and maintaining competence.

HCP described how they often had to “start from scratch” when a new case arrived, repeatedly reorienting themselves to meet the demands of each encounter. Care was often provided by HCP with limited experience in sexual assault cases, and workloads varied considerably across settings. As one nurse explained: It is challenging, because we are inexperienced; it’s not something we do every day. Each time we must familiarize ourselves with something new.” For some HCP, the primary challenge was managing several cases within a short time span, whilst others, particularly those working in rural SACs, described long intervals where weeks could pass between cases. In both situations, routines and skills were experienced as fragile. Despite limited institutional recognition, fragile routines, and variable workloads, HCP consistently emphasized their commitment to providing compassionate and competent care, underscoring a strong sense of professional purpose that was maintained under demanding conditions.

### Navigating medico-legal demands while providing care in vulnerable encounters

This theme illustrates the constant balancing act that HCP described between forensic accuracy and patients’ needs for dignity, autonomy, and safety. HCP emphasized that relational care and the establishment of trust were as crucial as medico-legal precision. In complex and often confusing situations, HCP had to navigate legal and medical requirements while simultaneously protecting patients’ sense of agency and emotional comfort. Careful forensic procedures, such as the collection of swabs and the completion of documentation using photos, diagrams, and detailed medical recording, were viewed as essential for securing both patients’ health and their legal rights. At the same time, HCP described forensic evidence collection as highly serious and technically demanding, with the work intensified by patients’ vulnerability and the time-pressure and often busy clinical environment. **This double responsibility**, being both technically precise and relationally attentive, was experienced as demanding, yet also meaningful, reflecting the complexity of providing care in situations of acute stress.

The tension between forensic demands and relational care became particularly visible during the clinical examination. HCP described how they first listened to the patient’s story and prepared them for each step of the examination before moving to the physical and gynaecological procedures, allowing the patient’s account to guide the encounter. Some phases were experienced as especially sensitive, such as when the patient was placed in the lithotomy position or when forensic sampling began. As one nurse noted: “If patients are going to break down, it is usually when the speculum goes in, when the forensic sampling starts – then you feel, now it is happening – now we are actually going to collect evidence.” These moments required heightened attentiveness and a deliberate, relational sensitivity. To foster safety, HCP emphasized non-judgemental presence, a calm tone of voice, gentle touch, and attentive body language, small relational gestures used consciously to counterbalance the invasiveness of forensic procedures. As one nurse reflected:

I think about our body language, that our body language makes them feel welcome and cared for (…) And it is also about how you adjust yourself in the room … you try to sit where it feels right. You read the room and signal that you are close.

Building on this relational sensitivity, HCP also emphasized how care involved an ongoing negotiation of power and control. Care was described as **balancing power and responsibility**, with HCP emphasizing the importance of supporting patients’ sense of control, for example by making it clear that they could stop or opt out of any part of the examination. HPC described prioritizing active listening and continuous validation throughout the entire process, from initial conversation to examination and documentation, as a way of supporting patients’ sense of control.

Voluntariness and consent were understood not only as a legal principle but also as an ethical anchor point in the encounter with the patient. Entering the most private and intimate aspects of a person’s body and experience also required HCP to navigate boundaries and intimacy. As reflected by one nurse: “It is kind of invasive what we are doing, so that is one of the challenges (…) We [HCP] get very close to them, that zone of untouchability, like we examine their bodies, step by step.” Or as expressed by another nurse: “It was so very intimate in a way, you are supposed to go around with that swab on them, around the nails and the hands. I think that part in the mouth, that was uncomfortable.”

Through this work, HCP aimed to **provide care on the patient’s terms** while simultaneously meeting medical and legal requirements. HCP described efforts to avoid reinforcing feelings of guilt or shame, for example through how questions were framed and how patients’ stories were acknowledged, even when fragmented, like this reflection from one of the nurses: “When you put those pieces together, it often turns into shame, the patients say: “I shouldn’t have” or “I shouldn’t have done that.” In response, I [HPC] often tell patients that nothing they are telling me is their fault. This balancing act extended beyond the physical examination and was most evident in the accompanying conversations. HCP described how asking about highly sensitive issues, sexual acts, medication use, or substances was often necessary for medical and forensic purposes, even when such information felt deeply private or potentially incriminating for the patient.

Several HCP described an awareness that their documentation might later be scrutinized in legal proceedings and expressed a perceived need for external forensic expertise to review and contextualize SAC findings. They also highlighted a tension between the legal emphasis on a free narrative and the clinical need for detailed, clarifying questions. As one participant explained, HCP risk “putting words into the patient’s mouth”, which may unintentionally complicate later investigative processes. Although medico-legal responsibility was described as shared, HCP emphasized different aspects of this role depending on their professional position, with physicians describing greater comfort than nurses with structured, and at times rather intrusive, questioning.

### The need to reset – Carrying the unsolved

This theme highlights the emotional weight of SAC work. HCP described how encounters stayed with them, with some stories “coming home with them”. One described the experience as “an emotional elastic band”. Beyond the ethical challenges noted earlier, this theme captures the ongoing work of emotional regulation, how **HCP “reset” themselves** to manage the residue of difficult encounters and sustain their ability to provide care.

Resetting, collegial support, and resilience strategies were described as essential for maintaining professional endurance. The work was both meaningful and taxing, and HCP emphasized the need to reset, especially when shifting rapidly between clinical roles or from private life into acute consultations. Several participants, particularly on-call physicians, described being summoned directly from other duties, sometimes involving routine or high-intensity clinical work, before immediately entering situations marked by trauma and vulnerability. As one of the physicians expressed it:

Yes, first it can be busy on call, you might come straight from an emergency C-section, and you’re stressed and tired, and then you must reset yourself. Then you go in and try to be as empathetic as you possibly can. To be there in the moment, but it’s not always that you manage, because your thoughts are elsewhere … It becomes very personal and asks a lot of questions … so that you can record this and get everything documented properly. So, you juggle back and forth between being a forensic and being a treating physician.

This reflection captures **the emotional transition** between markedly different clinical contexts and the need to **rapidly shift from one demanding situation to another**, while still providing care with presence and empathy.

This balance between the professional and the fellow human being, between the demands for accuracy and the need for presence, was described as a constant tension in everyday work. The structure of the SAC and the on-call system also shaped the character of the work. HCP emphasized that sexual assault cases could arise at any time, and that forensic sampling, conversations, and documentation were often time-consuming processes. In addition, the work required close collaboration and task-sharing between nurses and physicians, where clear communication and mutual support were crucial to ensure both medical quality and patient care. This required both flexibility and endurance. As one physician expressed it: “What I find most challenging, the worst is when you get the call-out, then I have to rearrange everything, but I find that it gets better once I get started.”

Even when the initial tension subsided once the work had begun, HCP described how the emotional impact often lingered after the SAC consultation had ended. Shifting between tasks and emotional states required not only flexibility in the moment but also ways of processing what remained unresolved. Beyond the immediate emotional strain, HCP described how the work frequently followed them afterwards in the form of persistent reflections, wondering whether they had overlooked something, failed to notice a detail, or missed an opportunity for follow-up. This also included concerns about what patients would face after leaving the SAC, particularly in situations where the assault had occurred in the patient’s own home or involved someone living nearby. The knowledge that some patients were returning directly to environments associated with the assault was described as especially troubling. This sense of carrying “unfinished business” was described as particularly demanding, as one nurse expressed it: “It is the unresolved that we carry with us.”

To manage the combined strain, whether from heavy workloads or long intervals between cases, HCP emphasized **the importance of support and recognition**. Community, humour, and spaces for reflection were described as vital resources. Collegial support was considered crucial for sustaining them in the work, often compensating for the absence of more formalised structures for supervision and support. As one nurse put it:

This weekend I had four cases, and it takes a toll, but it’s kind of like – then it’s good to have good colleagues, it makes it a bit easier, and we get through it. I must admit it’s exhausting, but it’s motivating.

The staff room was described as an important arena for support, processing, and professional fellowship. As one nurse put it: “The staff room is where it happens, where we meet and talk, and where we share experiences.” Alongside collective spaces for reflection, many also developed personal strategies to disengage from work after a shift. Some sought solitude in nature, whilst others used a designated physical space, referred to by colleagues as a “screaming corner”, to release accumulated tension. These individual and shared practices were described as essential ways of resetting, allowing staff to recover emotionally before returning to work or home life. HCP emphasized that working at the SAC meant constantly **juggling different professional roles** and emotional states, often with limited time to adjust. The work therefore required not only clinical competence but also endurance, flexibility, and emotional resilience. As one nurse expressed it: “You need to have the energy to endure other patients’ rough times, while also having something left for your own family – you need a plan for how to put the job to one side.” Through these accounts, it became clear that the ability to “reset”, both collectively and individually, was vital for sustaining empathy and professional presence over time.

## Discussion

This study offers insight into HCP’s experiences of working in SACs and identified three interrelated themes that shed light on how emotional, relational, and institutional demands are challenged in practice. **The first theme** highlights how HCP experience their work as deeply meaningful and motivated by the difference that they can make for patients in a low-status field of care, despite limited training and a lack of institutional recognition. Consistent with previous research showing strong intrinsic motivation among professionals working with sexual assault survivors,^2,12,37^ HCP in this study described their work as *contributing to something greater*. Witnessing that their care made a tangible difference for patients, and engaging with individual patient stories, emerged as central sources of professional meaning, echoing studies that demonstrated that perceived patient progress is a key driver of motivation in trauma care.^3,32,35,44,45^

Although research shows that most assaults are committed by someone known to the victim, rape myths, particularly the idea of the stranger perpetrator, may continue to shape forensic practice, despite the good intentions of individual practitioners.^28^ At the same time, our findings indicate that HCP, like the broader public, may be influenced by culturally dominant “real rape” and “real victim” narratives,^46,47^ even when striving to provide equitable and trauma-informed care, potentially shaping clinical judgement and interpretations of patients accounts.^3,45^ Encounters were described as particularly meaningful when the assault appeared “serious” and clearly recognizable, whereas cases involving fragmented memories, uncertainty, or only a vague sense that “something was wrong” were experienced as more emotionally and practically challenging. These observations mirror well-documented expectations of the “real victim”, someone who remembers the event clearly, seeks help promptly, presents a coherent narrative, displays visible bruises indicating violence, and shows credible trauma reactions. When patients’ accounts deviated from this narrative, they could unintentionally evoke less immediate emotional resonance, not because HCP doubted their credibility but because such narratives were harder to situate within familiar cultural models of sexual assault.^47–49^

This tension corresponds with research showing that many survivors themselves struggle to label their experiences as sexual assault when incidents do not align with dominant expectations.^3,46,50,51^ Studies demonstrate that assaults involving alcohol, prior familiarity with the perpetrator, or the absence of overt physical force are frequently framed as “miscommunication”. Fragmented memories, self-blame, and uncertainty about consent further constitute major barriers to help-seeking.^48^ When such narratives enter clinical encounters, HCP must navigate the absence of clear details by maintaining a commitment to unconditional care. Our findings suggest that the interplay between cultural rape myths, survivor uncertainty, and HCP’s emotional responsibility may shape the everyday moral and emotional labour at SACs.

Importantly, this dynamic is not unique to care at SACs but reflects broader tendencies in healthcare to privilege conditions with clear, observable signs, often understood as “real disease”, over subjective or ambiguous presentations.^52^ Research shows that clinicians may find it more satisfying and less demanding to engage with conditions that map neatly onto biomedical categories, whereas diffuse or “invisible” conditions can provoke greater emotional strain and uncertainty.^53,54^ In this context, the stronger emotional engagement described in clearly defined “serious” assault cases may reflect a broader professional inclination towards narratives that feel structured, legible, and medically coherent. When patients present with uncertainty or absence of visible injury, their accounts may inadvertently resemble other clinical presentations that challenge legitimacy and emotional engagement in healthcare.^54^

Alongside this strong sense of purpose, HCP reported limited formal training, unclear organizational structures, and a perceived lack of institutional recognition. This tension between high emotional investment and limited systemic support mirrors findings from emergency and forensic nursing litterture,^1,37^ where work is often described as mission-driven yet institutionally invisible. In line with our findings, HCP have been shown to derive both personal and professional fulfilment from their role, experiencing meaning, pride, and empathic satisfaction when patients show relief, and when their work contributes to justice and societal benefit.^21^

**The second theme** illustrates how HCP continuously navigate the dual nature of their role, balancing medico-legal requirements related to documentation, evidence preservation, and forensic examination with compassionate, person-centred care. This “double responsibility” required clinicians to balance power and vulnerability, acting both as the authority responsible for evidence collection and as the relational partner attending to patients’ emotional needs. Similar tensions have been reported internationally,^21^ particularly in contexts such as the Norwegian system, where HCP serve both as caregivers and forensic evidence collectors. In the present study, clinicians framed this balancing act not as a conflict between two roles but as an integrated professional responsibility. Rather than choosing between accuracy and compassion, they worked continuously to uphold both, suggesting that SAC clinicians practice a highly advanced form of relational–forensic competence that remains insufficiently recognized in policy and training structures.^32,55^

At the same time, this integrated role raises important implications for legal safeguards, as complete neutrality is difficult to achieve when the same clinician both delivers care and conducts forensic procedures. Several HCP noted that their documentation may later be presented in court, and that external forensic experts are therefore needed to review and contextualize SAC findings to ensure procedural fairness. The professional differences described in the results further underscore this tension. Whilst physicians appeared more comfortable with rather intrusive but structured questioning, nurses more often emphasized the ethical responsibility to avoid unintentionally steering patients’ accounts. This concern resonates with Martinsen’s^56^ interpretation of Løgstrup’s notion of the zone of untouchability, which emphasizes the ethical obligation to protect the patient’s experiential and narrative boundaries when exercising professional authority. This highlights how medico-legal responsibility is enacted differently across professional roles, even within the same clinical encounter. In addition, clinicians reflected on tensions between the medically necessary detailed questioning and the police’s reliance on a free narrative, implying that clinical questioning may inadvertently influence later statements.

These concerns resonate with Yesodharan et al.’s ^57^ argument that communication techniques may unintentionally shape medico-legal histories, thereby underscoring the need for specialized training in forensic interviewing. At the same time, Schmitt et al.^58^ have shown that prosecutors often regard SANEs’ involvement as improving documentation, injury identification, trial preparation, and ultimately evidentiary credibility in court.

Importantly, some HCP described a sense of professional validation when their careful documentation contributed to legal outcomes, highlighting how the dual role may foster a subtle advocacy stance towards the patient. Although such investment reflects a strong ethical commitment, it also underscores why clear forensic safeguards and external review mechanisms remain essential to protect both victims and defendants. This awareness of responsibility towards both patients and potential suspects contributed to clinicians’ anxiety around documentation and questioning, as errors or unintended bias could have serious legal consequences for individuals who were later identified as alleged perpetrators. Taken together, these findings suggest that SAC clinicians perform a complex medico-legal role that both requires robust safeguards and, when appropriately supported, has the potential to strengthen, rather than undermine, legal processes.

**The third theme** captures the emotional residue that clinicians carry with them after encounters with victims of sexual assault.^37,59,60^ HCP described the emotional labour involved in shifting between intense SAC work and routine clinical duties – a process they referred to as “resetting themselves”. Unresolved cases and lingering impressions often extended beyond the clinical setting, shaping how clinicians carried their work with them after shifts and, at times, contributing to emotional strain that could affect sustained engagement in this field. Collegial support emerged as essential for managing this emotional residue. Consistent with previous research, such as that by Rudolfsson and Punzi,^64^ clinicians noted that collegial support was crucial for buffering emotional strain and preventing secondary traumatic stress. Informal conversations in staff rooms or brief exchanges between tasks provided opportunities to pause, share difficult experiences, and receive understanding from colleagues who were familiar with the work. These spontaneous, low-threshold interactions offered immediate validation and relief, supporting clinicians in collectively resetting as they moved between demanding forensic tasks and ordinary clinical responsibilities.

These accounts resonate with research from emergency care, acute psychiatric inpatient settings, and trauma-exposed healthcare environments, where both structured and informal opportunities for reflection, debriefing, and collegial support have been identified as essential protective factors against secondary traumatic stress and vicarious trauma.^37,61^ Studies consistently show that collegial support, supportive leadership, and having spaces (formal or informal) in which to process emotionally demanding encounters are associated with lower levels of stress and greater emotional resilience. Likewise, scoping reviews highlight that group-based and relational interventions tend to be particularly effective for trauma-exposed professionals, as they support shared meaning-making and reduce the sense of carrying the emotional burden alone.^62,63^

In this context, staff room conversations and “meetings in between” were described as integral, rather than incidental, to sustaining emotionally demanding practice.^64^ This aligns with Rudolfsson and Punzi’s findings,^64^ that female HCP experience care after sexual assault as personally affecting, heightening awareness of violence against women and reinforcing the need for collegial support.^64^ Whether grounded in humour, shared reflection, or mutual reassurance, these interactions played a central role in maintaining professional endurance and enabling the emotional resetting necessary to sustain presence, empathy, and clinical accuracy over time. This is consistent with findings from a recent qualitative systematic review,^21^ which shows that HCP working with sexual assault victims rely heavily on informal collegial support and shared reflection in the absence of structured supervision, while national mapping in Norway^1^ similarly indicates that such informal practices often compensate for limited access to formalized professional guidance.

## Strengths and limitations

This study has several methodological strengths. Participants were recruited from both rural and urban SACs, and the sample included a balanced representation of physicians and nurses, allowing for exploration of role-specific perspectives. Focus groups facilitated rich discussions and allowed participants to build on each other’s reflections, contributing to the development of shared understandings.^39^ At the same time, the group context may have shaped which perspectives were expressed. Some participants may have been less inclined to voice divergent or critical views, particularly in the presence of colleagues. A limitation of this study is the lack of direct patient and public involvement in its design, although input from clinical stakeholders was incorporated. While survivor perspectives were not included in this specific study, user involvement was integrated in related patient studies within the broader doctoral project.^3,45^ In addition, the study was informed by meetings and presentations with SAC leaders, who also have clinical experience, during the planning phase, allowing for input from clinical practice. The interview guide was also presented and discussed with SAC leaders, further strengthening its clinical relevance.

Importantly, the two researchers, SH and MK, collecting the data were external to the SAC setting, which may have supported an analytic stance with professional distance from the field and reduced pressure to present services or practices (HCP) in a favourable light. A potential limitation relates to the involvement of a SAC clinician in the research team. The initiator of the project, CTH, is a physician at SAC and conceived the study to address important knowledge gaps in the field. To support openness in the interviews and analytic reflexivity, CTH did not participate in the staff group interviews, and was not involved in the coding process. However, she contributed to later stages of analysis, including interpretation and synthesis. While this contributed important clinical and multidisciplinary perspectives to the research team, her involvement may also have shaped the interpretation of the data. The first author maintained primary responsibility for the analytic process and ensured that the findings were grounded in the participants’ accounts.

To enhance the quality and trustworthiness of the analysis, we drew on Braun and Clarke’s guidance for high-quality reflexive thematic analysis.^38,41^ Coding was conducted iteratively, and analytic decisions were developed through ongoing discussions within the research team, where preliminary codes and themes were revisited and refined in relation to the data extracts. Reflexivity was addressed through continuous consideration of how the researchers’ disciplinary and clinical backgrounds may have shaped both data collection and interpretation. In line with this approach, analytic rigour was understood as grounded in reflexivity, coherence, and richness and variation in the dataset, rather than in procedural criteria such as data saturation or coding reliability.^38^ Findings were co-constructed, acknowledging that researchers’ backgrounds and assumptions shape data interpretation. Reflexivity was addressed through continuous analytic discussions within the research team and by maintaining an audit trail of codes, themes, and analytic decisions.^38^ The iterative movement between data, analysis, and interpretation allowed for critical reflection and refinement of themes, ensuring that findings remained grounded in participants’ accounts.^38^

More broadly, qualitative research has emphasised the importance of depth, meaning, and variation in data, rather than numerical adequacy.^65^ Saturation has been conceptualised in multiple ways across qualitative methodologies, with considerable variation in how it is understood and applied.^66^ While it has often been treated as a universal marker of quality, this assumption has been increasingly questioned, particularly within reflexive thematic analysis.^38^ However, some limitations should be considered when interpreting these findings. Voluntary participation may have introduced self-selection bias, with HCP particularly engaged in care following a sexual assault care being more likely to participate. The study was conducted in a single national context with a relatively small sample, which may limit transferability.

## Conclusions

This study highlights how HCP at Norwegian SACs navigate the clinical, medico-legal, and emotional demands of care following a sexual assault. The findings demonstrate the dual responsibility of providing forensic and therapeutic care in situations of acute vulnerability, while safeguarding patients’ dignity, safety, and autonomy. Despite limited training and structural constraints, HCP derived meaning from their work and relied on collegial support to sustain professional practice. Together, the findings point to the need for targeted training, organizational support, and ethical sensitivity to ensure the provision of high-quality, person-centred trauma-informed careFigure 1.

**Figure 1.**
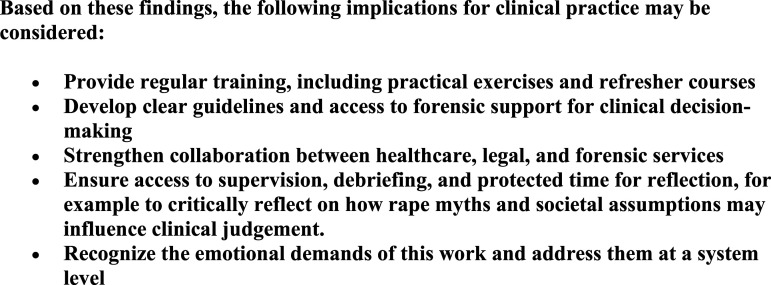
Visualize implications.

## Implications for clinical practice

The findings underscore the complex and emotionally demanding nature of sexual assault care and the substantial responsibility placed on HCP working at SACs. Although strong professional motivation was evident, reliance on individual commitment alone is insufficient to sustain the provision of high-quality, trauma-informed care over time. The results highlight the need for organizational structures that acknowledge and support the emotional, ethical, and medico-legal complexity of this work. Taken together, the findings suggest that these challenges are not only individual but reflect broader structural and cultural conditions shaping SAC practice.

First, the study points to the importance of structured and ongoing training tailored to the realities of care after sexual assault. HCP require education that integrates trauma-informed, person-centred practice with forensic competence, including documentation of injuries, specimen collection, communication, and not the least awareness of how clinical questioning may influence legal processes. Such training is particularly important given the irregular nature of SAC work and the risk of skill erosion during periods with few cases. Training should also include knowledge about how severe trauma can affect memory, narrative coherence, and emotional expression, and how this may influence how patients present their experiences and how HPC can prevent PTSD.^67–70^ In addition, training should address common rape myths^46^ and societal understandings of sexual violence, and how these may shape clinical judgement and perceptions of “credible” cases.

Second, the dual role of SAC clinicians as caregivers and forensic actors calls for clear organizational frameworks and safeguards. Access to external forensic review, clear interagency guidelines, and collaboration with legal actors may help protect both patients’ and defendants’ rights while reducing clinicians’ anxiety related to neutrality and potential error. These findings also raise questions about the inherent tension between care and investigation, suggesting a need for clearer structural support in navigating these potentially conflicting demands.^28^

Finally, the emotional labour that is inherent within the care at SACs requires systematic attention to sustainability and staff wellbeing. Collegial support and informal spaces for reflection emerged as essential resources and should be recognized as integral to care delivery rather than as optional coping strategies. However, these findings also point to a reliance on informal and individual coping strategies in the absence of more structured and system-level support.

## Data Availability

Due to the sensitive nature of the qualitative interview data and the need to protect participant confidentiality, the datasets generated and/or analysed during the current study are not publicly available.*
